# Real world data of a veterinary teaching hospital in Japan: a pilot survey of prescribed medicines

**DOI:** 10.1136/vetreco-2016-000218

**Published:** 2017-09-26

**Authors:** Noriko Tanaka, Tsuyoshi Takizawa, Nao Miyamoto, Shinji Funayama, Ryo Tanaka, Syozo Okano, Toshio Iwasaki

**Affiliations:** 1 Department of Pharmaceutical Sciences, Nihon Pharmaceutical University, Kitaadachi-gun, Japan; 2 Department of Pharmaceutical Sciences, Chiba Institute of Science Shiomi-cho Chiba, Chiba, Japan; 3 Global Clinical Development, PPD-SNBL K K, Tokyo, Chuo-ku, Japan; 4 Department of Veterinary Surgery, School of Veterinary Medicine, Tokyo University of Agriculture and Technology, Tokyo, Japan; 5 Department of Small Animal Surgery, Veterinary Teaching Hospital, School of Veterinary Medicine, Kitasato University, Aomori, Japan

**Keywords:** electronic database, digital accounting system, surveillance of prescribed medicines, companion animals, veterinary teaching hospital

## Abstract

The prescription data from a digital accounting system of a veterinary teaching hospital collected between 2008 and 2011 in Japan were downloaded, stored in a database and analysed using a statistical analysis software, SAS. Seventy-six per cent of all prescriptions were drugs approved for human beings. The most frequently prescribed category was ‘Agents against pathogenic organisms’, such as antibiotics and chemotherapeutic agents, followed by ‘Cardiovascular agents’. Seventy-five per cent of prescribed oral formulations in the category ‘Agents against pathogenic organisms’ were drugs approved for human beings, while 78 per cent of the injectable prescriptions were those for veterinary. A total of 36 oral antipathogenic products were prescribed, and among them amoxicillin was prescribed the most, followed by cephalexin for human beings and enrofloxacin for veterinary. The pattern of cyclosporin prescription, which is the most prescribed product other than ‘Agents against pathogenic organisms’, was surveyed. The capsule formulation was primarily used for dogs, while oral solutions were preferably used for cats. This pilot study is the first analytical data of real prescription in hospitals in Japan and one of the longest surveys in veterinary world.

## Introduction 

Therapies applied to older companion animals have become increasingly frequent based on the amount of health insurance payments claimed by owners of companion animals reported by an insurance company in Japan (Statistics on household animal diseases in ‘White Paper on Household Animals 2013’, 2013, Anicom Holdings, Tokyo, *in Japanese*).^1^ Age-related disorders in companion animals, such as malignancies, cardiovascular diseases, obesity and diabetes, increase the need for medical products that are different from the currently approved for veterinary. This urgent need for a variety of pharmaceuticals promotes the off-label use of pharmaceuticals intended for human use. Prescription data regarding off-label use of human medicinal products have never been systematically investigated in Japan, and there are no legal obligations to report any results, even those related to possible safety issues caused by the drugs. No postmarketing surveillance exists. Reports, publications or consultations by veterinarians, on their own accord, are the only methods to share information regarding the off-label use of drugs approved for human beings. According to a survey of secondary referral veterinary hospitals in Japan, more than 90 per cent of prescribed medicines are for human beings.^2^ Although a digital health record system is available in several Japanese veterinary teaching hospitals, almost all of the patient records are still stored as handwritten documents except for the records that are necessary for accounting purposes. However, all the teaching hospitals have electronic accounting systems in which daily records of medicinal products prescriptions are stored electronically.

Prescription surveys have been conducted in veterinary hospitals in Norway,^3^ Finland,^4 5^ Switzerland,^6^ Spain,^7^ the UK^8 9^ and the USA.^10 11^ These surveys were conducted using data from electronic medical records as well as paper documents recording the prescriptions. Radford and others conducted a survey using the prescription data from ‘The Small Animal Veterinary Surveillance Network’.^12^ The periods of time covered by these surveys are relatively limited, from four weeks to two years. There is, however, no prescription surveillance report from Japan. In the present study, prescription surveillance was conducted using electronic animal health records from the accounting system that was most widely distributed in veterinary practices and teaching hospitals in Japan. This is the first report of a survey of medical product prescriptions in a Japanese veterinary teaching hospital using real data from electronic records.

## Materials and methods

### Selection criteria of hospital

One out of 16 veterinary teaching hospitals in Japan was selected based on the results of the questionnaire survey.^2^ The secondary referral hospital for companion animals has a digital accounting system, which is widely used in Japan having a support of maintenance contract that operates to download data by remote control.

### Prescription records

Electronic prescription records were downloaded from a hospital accounting system, Anicom receptor (Anicom Pafe, Tokyo), with the consent of the hospital and Anicom Pafe, which has a maintenance contract for the system with a veterinary teaching hospital located in a suburb of Tokyo. The process of downloading the data was handled by a system engineer whose work was conducted under a contract. The downloaded records included the following information: item number, treatment such as a product name, prescription date, usage, dosage, patient identification number (ID number), animal species, breeds, sex, weight, age, serial number and health record number. Personal data such as the patient owners’ names, any information or ID numbers related to the owners, or the patients’ names were not downloaded. The electronic file containing the downloaded data was sent to the author with a password. The data from July 1, 2008 to November 30, 2011 were analysed in the present study.

### Product information

The downloaded treatment records included medicinal products and medical treatment procedures, such as type of injection medical devices and so on, but the treatments other than medicinal products were excluded; diagnostic agents and/or supplements were excluded from the present analysis. Each prescribed product shown as a brand name registered in the accounting system was corrected based on the brand name of the package inserts. Simple misspellings, which were easily identified as a mismatch with the brand name, were corrected. The generic names of the active ingredients and a Japan Standard Commodity Classification Number for each product were added for each product (Ministry of Internal Affairs and Communications, Statistics Bureau, 2012, available at http://www.stat.go.jp/english/index/seido/8.htm). The generic name of imported products was added according to ‘Drugs@FDA’ or ‘Animal Drugs@FDA’ from the homepage of the US Food and Drug Administration (available at http://www.accessdata.fda.gov/scripts/cder/drugsatfda/index.cfm or http://www.accessdata.fda.gov/scripts/animaldrugsatfda/) or the homepage of the European Medical Agency (available at http://www.ema.europa.eu/ema/index.jsp?curl=/pages/home/Home_Page.jsp&jsenabled=true). In the case of products that were no longer commercially available, the brand name and generic name that are used in the market were added. In principle, according to the Japan Standard Commodity Classification (Ministry of Internal Affairs and Communications, Statistics Bureau, available at http://www.stat.go.jp/english/index/seido/8.htm), the classification of products was performed and the names of pharmaceutical therapeutic categories were used, but minor modifications were made because of the differences between agents used for human beings and those used for veterinary. The drugs and related commodities were divided into pharmaceutical therapeutic categories. Products having multiple indications with more than two classification numbers were grouped based on an interview with the clinicians at the hospital.

### Data analysis

The prescription records and the product name file were imported as data sets into SAS V.9.3 software (SAS Institute Japan, Tokyo, Japan). Match-merging the two data sets by treatment attached brand name, generic name and classification code to the prescription records. Frequency distributions from the prescription data were counted.

### Guidance

As routinely collected health data were used in the present study, RECORD items were referred.^13^

## Results

**Figure 1:**
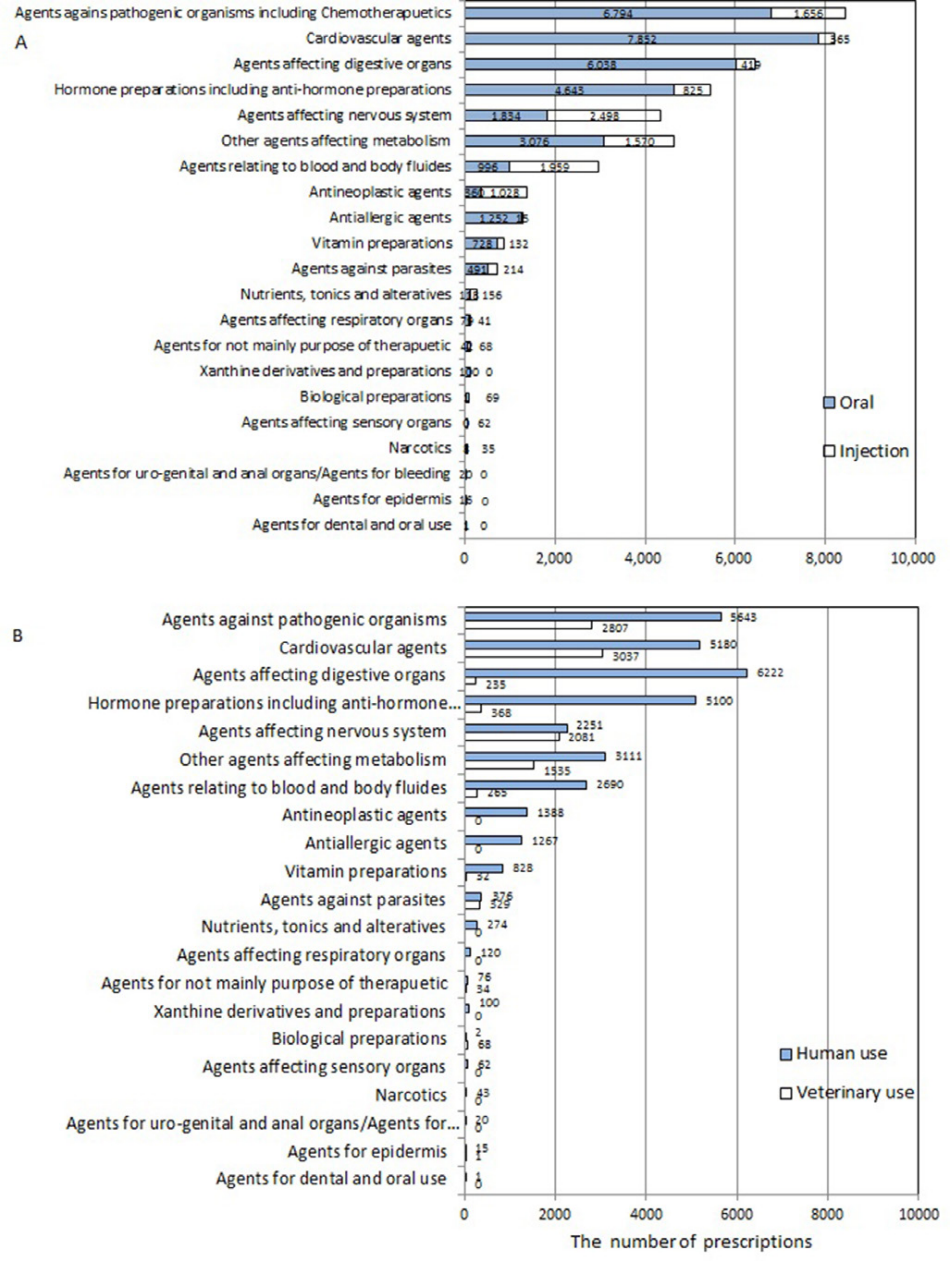
The number of prescriptions of pharmaceutical subcategories between July 1, 2008 and November 30, 2011. The number shown in the graph indicates the number of prescriptions in each category. (a) Prescription numbers of oral and injectable forms. The open column represents the injectable form, and the closed column represents the oral form. Oral forms were prescribed more frequently than the injectable forms at the hospital. (b) Prescription numbers of human and veterinary uses. The open column represents human use, and the closed column represented veterinary use. There was a tendency to prescribe products for human use in all subcategories with the exception of ‘Agents affecting the nervous system and sensory organs’, ‘Immunosuppressive agents’ and ‘Agents against parasites’.

The total number of included animals was 4833, and the data were collected for the study period from a hospital located in a Tokyo suburb (the number of consulted patients and the ratio of each species are shown in table 1). The number of dogs was fivefold larger than that of cats. Included species other than dogs and cats constituted less than one per cent, and horses from the university equestrian club were also included. The total number of prescriptions was 45,993 during this period, and 34,949 out of the 45,993 (76.0 per cent) prescriptions were for medical products approved for human beings (table 2). The numbers and ratios of oral or injectable products for human beings and veterinary are also shown in table 2. The ratio of injectable drugs for veterinary versus total was 8.2 per cent, but the ratio versus total of injectable drugs was 33.9 per cent (3767 out of 11,119). In the case of oral products, the ratio was 16.0 per cent versus total products, but 20.9 per cent (7277 out of 34,874) versus total of oral products. Overall, the prescription ratios of products developed for human use were much higher than those for veterinary use. Figure 1 indicates the number of prescribed products in each pharmaceutical subcategory of medicines for human and veterinary uses. The highest number of prescribed drugs belonged to the therapeutic category ‘Agents against pathogenic organisms apart from anti-parasites’, followed by ‘Cardiovascular agents’ and ‘Agents affecting digestive organs’. Among the products specifically developed for veterinary use, the highest number of prescribed drugs also belonged to the category ‘Agents against pathogenic organisms apart from anti-parasites’, followed by ‘Cardiovascular agents’. The number of prescriptions in the category ‘Agents against pathogenic organisms apart from anti-parasites’ was almost the same as that for ‘Cardiovascular agents’ in the hospital that provided cardiovascular consultations for outpatients.

**Table 1:** The number of consulted patients during the time period from July 1, 2008 to November 30, 2011

Total*	Dogs	Cats	Others†
4833 (100)‡	4037 (83.5)	785 (16.2)	11 (0.2)

*The number of patients was determined by ID numbers.

†Horses of the college riding club and pigs for veterinary practice.

‡The ratios of the total in parentheses.

**Table 2:** Prescription of oral and injectable products developed for human and veterinary preparations

Dosage form	Number of prescriptions (%)*		
	Human and veterinary preparations	Human preparations	Veterinary preparations
Oral	34,874 (76.0)	27,597 (60.0)	7277 (15.8)
Injectable	11,119 (24.2)	7352 (16.0)	3767 (8.2)
Total	45,993 (100)	34,949 (76.0)	11,044 (24.0)

*The ratio of total prescription in parentheses.

**Figure 2:**
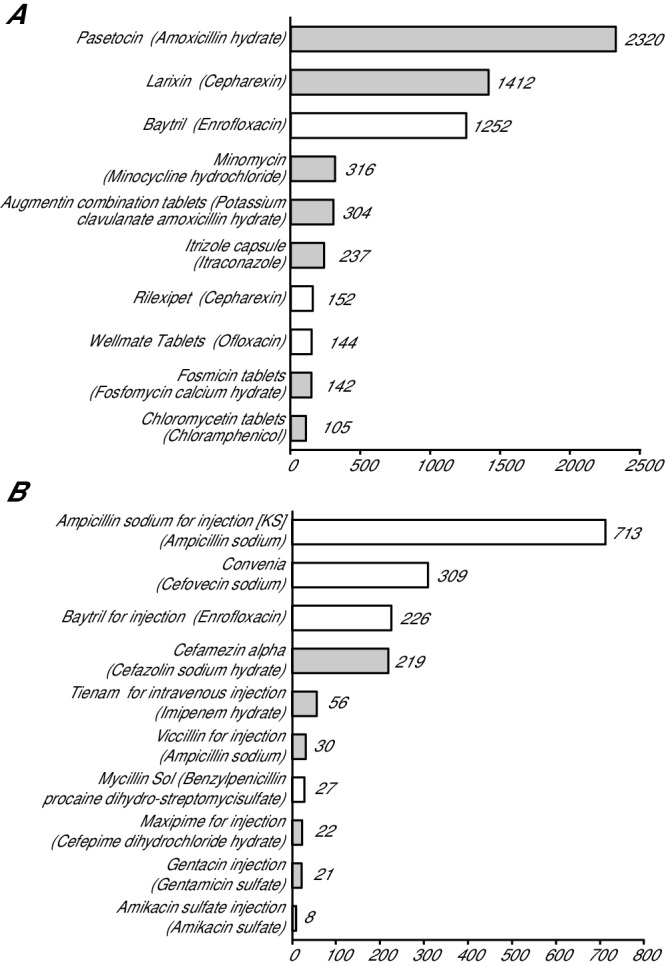
The top 10 products of ‘Agents against pathogenic organisms’: (a) oral products and (b) injectable products. The number on the graph indicates the prescription number of each product. The closed columns are the products for human preparations, and the open columns are the products for veterinary preparations. Generic names are shown in parentheses.

The total number of prescriptions of oral and injectable drugs in the category ‘Agents against pathogenic organisms’ was 8450; 6794 were for oral formulations and 1656 were for injectable formulations. Seventy-five per cent of the prescriptions for oral formulations (5095 out of 6794) were for products developed for human use. Conversely, 78 per cent of prescriptions for injectable drugs (1292 out of 1656) were for products developed for veterinary use. The top 10 most frequently prescribed products are shown in figure 2 as follows: oral products are shown in (a), and injectable products in the category ‘Agent against pathogenic organisms’ are shown in (b). A total of 36 oral products were prescribed, and among them amoxicillin was prescribed the most frequently, followed by cephalexin (developed for human use), enrofloxacin (developed for veterinary use) and minocycline hydrochloride (developed for human use). Enrofloxacin was the most frequently prescribed product originally developed for veterinary use. Oral cephalexin developed for veterinary use was the seventh most frequently prescribed product. The number of prescriptions of each therapeutic class in the category ‘Agents against pathogenic organisms’ originally intended for human use is shown in table 3. In the category ‘Agents against pathogenic organisms’, penicillins, including amoxicillin, were the most frequently prescribed antimicrobial class of drugs among the beta-lactams. First-generation cephems of the beta-lactam group were the most frequently prescribed. In the case of quinolones and new quinolones, more than 99 per cent of the prescribed drugs (789 out of 793) were developed for veterinary use.

**Table 3:** The number of prescribed products for animals within different antimicrobial classes

Antimicrobial class	Number of prescriptions				
	Total	Human preparations		Veterinary preparations	
		Oral	Injectable	Oral	Injectable
Beta-lactams					
Penicillins	1885	1456	4	0	425
Cephalosporins	1357	982	105	91	179
Penems	44	44	0	0	0
Carbapenems	36	0	36	0	0
Quinolones and new quinolones	793	4	0	693	96
Tetracyclines	261	261	0	0	0
Chloramphenicols	55	52	0	0	3
Macrolide	54	52	2	0	0
Lincomycins	54	20	0	34	0
Fosfomycins	38	38	0	0	0
Aminoglycosides	38	0	11	0	27
Sulfonamides	10	9	1	0	0
Glycopeptides	1	1	0	0	0
Total	4626	2919	159	818	730

The most prescribed veterinary product, other than quinolones, was Atopica capsules, in which the active ingredient is cyclosporin. Compared with the above ‘Agents against pathogenic organisms’, cyclosporin would typically be prescribed continuously because of its indication for a chronic disorder. In order to analyse sequential prescription, cyclosporin prescription was further surveyed. In addition to Atopica capsules, two oral cyclosporin products, Sandimmune oral solution 10% and Neoral oral solution 10%, both of which were developed for human use, were also prescribed. Among them, Atopica capsules were the most frequently used cyclosporin product, as shown in table 4. Atopica capsules were the most frequently prescribed drugs for dogs among the three products, and the prescription rate was 93.8 per cent (744 out of 793), while the oral solutions, Sandimmune or Neoral, were used at a rate of only 6.2 per cent (49 out of 793). Conversely, oral solutions of cyclosporin products, such as Sandimmune and Neoral, were more frequently prescribed for cats. Thirty-three out of 98 prescriptions, that is, 33.6 per cent, were for oral solutions, Sandimmune or Neoral. No oral solutions of cyclosporin products were commercially available for veterinary use during the survey period.

**Table 4:** Species differences in prescription rates of oral cyclosporin products

Species	Number of prescriptions (%)		
	Total	Capsule*	Oral solution†
Dogs	793 (100)	744 (93.8)	49 (6.2)
Cats	98 (100)	65 (66.3)	33 (33.6)

*The total prescription number of Atopica capsules with any contents.

†The number of prescriptions of Sandimmune oral solution 10% and that of Neoral oral solution 10% were added together.

## Discussion

Several surveillance studies were conducted and reported in Norway,^3 14^ Finland,^4 5^ the UK,^8 9^ Sweden,^15^ Switzerland,^6^ Spain^7^ and the USA^10 11^ using electronic records, prescription paper records or internet databases. A four-week survey of prescriptions was conducted in Norway based on the prescriptions filled at 269 pharmacies, and it reported that 43.3 per cent of the total prescriptions were for products that were approved for human use, but 71 per cent of the prescriptions were approved for companion animals.^3^ In Finland, only 17 per cent of products prescribed were approved for human use.^5^ These differences may be caused by either different regulations, differences in approved products in each country, different survey periods, different animal populations and different therapy choices for aged companion animals. Products approved for human use are more frequently prescribed for companion animals in several countries, including Japan. We surveyed the products supplied and recorded in an accounting system of a veterinary hospital and found that 86 per cent of the products were developed for human use.^16^ In the present study, we established a method to download electronic records from an accounting system and constructed a database using SAS Enterprise Guide, which is a software that is widely used for clinical trials for medicinal products for human use. Data from the accounting system of one veterinary teaching hospital were surveyed in the present pilot study for the first time in Japan: the surveillance term was from 2008 to 2011. The duration is much longer than the above-mentioned studies outside Japan. Prescription survey based on the data from other hospitals is in progress. Currently, antimicrobial consumption survey based on national sales data,^17^ and online survey were reported.^18^ The use of current data mining tools can give new aspects of clinical real data timely.

To date, the number of prescription surveillance studies is still limited. Among the published studies, antimicrobial prescriptions corresponding to the present category ‘Agents against pathogenic organisms’ were the focus.^5 6 12^ Antimicrobial use guideline and antimicrobial use are also investigated.^19^ The contribution of antimicrobial use to the development of drug resistance has increased the awareness of antimicrobial prescription patterns among the veterinary as well as the human health communities. The use of electronic records may help in the handling of prescription surveillance, both in directly monitoring use and in promptly analysing trends. In the present study, antimicrobials were easily selected from all electronic records using code numbers based on the Japan Standard Commodity Classification Number system. As shown in figure 2 and table 3, ampicillin was preferably prescribed, followed by cephalexin and enrofloxacin, in the hospital. These results are similar to the results reported by Wayne and others in a US teaching hospital. They found that the most frequently prescribed antimicrobials were ampicillin-clavulanate, followed by cephalexin or cefazolin, enrofloxacin, and amoxicillin or ampicillin.^11^ In Japan, the Japanese Veterinary Antimicrobial Resistance Monitoring System (JVARM), established in 1999, monitors the occurrence of antimicrobial resistance in bacteria ‘in food-producing animals’ and monitors the consumption of antimicrobials for animal use. The animal species assessed by JVARM are restricted to ‘food-producing animals’, and companion animals are not monitored by JVARM. A prompt and accurate monitoring system should be developed also for companion animals. The present study indicated that antimicrobials used for companion animals can be monitored using database as follows. The most prescribed amoxicillin was Pasetocin, a product approved for human use (Kyowa Hakko Kirin licensed by GlaxoSmithKline), although an amoxicillin product approved for veterinary use, Bacillion tablet 100 (Meiji Seika Pharma), exists. The prescription of quinolones was the second most among antimicrobials and the most among drugs approved for veterinary. The increase of quinolones in prescriptions for dogs in Norway was also reported.^14^ Although year-by-year change in prescriptions was not shown in the present pilot study, the data will be available in subsequent studies.

Other than ‘Agent against pathogenic organisms’, cyclosporin, of which indication was chronic disorder, was the most prescribed among veterinary products. We evaluated the species differences in choice of formulations exist: the oral solutions were preferable for cats. According to Plumb’s Veterinary Drug Handbook, the injectable form of Sandimmune is recommended when the oral formulation is difficult to administer to cats. Oral solutions have been developed for many medicinal products but are still limited.

Although the combination pattern or change per year of a certain product prescription can be easily analysed using the present database (data not shown), diagnoses, symptoms, findings, clinical laboratory examination data and other information were regretfully not included in the data downloaded from the accounting system. In addition, patient information data, such as weight and age, were not fully inputted by veterinarians even though data-item box exists. Health records were stored only in paper form, indicating that it may be difficult to select specific consultation cases for retrospective studies. Subsequently, data merge with data of another teaching hospital is in progress. The more data from other hospitals joining the surveillance study can introduce the more useful and advanced analysis. If more health records become electronic, an expanded database could be applied for more precise retrospective studies, and cohort studies as well as the establishment of guidelines and assessments of adherence to guidelines.
